# Separable roles for RanGTP in nuclear and ciliary trafficking of a kinesin-2 subunit

**DOI:** 10.1074/jbc.RA119.010936

**Published:** 2020-12-03

**Authors:** Shengping Huang, Larissa L. Dougherty, Prachee Avasthi

**Affiliations:** 1 Department of Ophthalmology, University of Kansas Medical Center, Kansas City, Kansas, USA; 2 Department of Anatomy and Cell Biology, University of Kansas Medical Center, Kansas City, Kansas, USA; 3 Department of Biochemistry and Cell Biology, Geisel School of Medicine at Dartmouth College, Hanover, New Hampshire, USA

**Keywords:** RanGTP, kinesin-2, KAP3, nuclear import, ciliary targeting, *Chlamydomonas*, ARM6–9, armadillo-repeat region 6–9, BSA, bovine serum albumin, DMEM, Dulbecco's modified Eagle's medium, DMSO, dimethyl sulfoxide, EGFP, enhanced GFP, HA, hemagglutinin, hnRNP A1, heterogenous nuclear ribonucleoprotein A1, hTERT-RPE, human telomerase reverse transcriptase-retinal pigment epithelial, IF, immunofluorescence, IFT, intraflagellar transport, IPZ, importazole, KAP3, kinesin-associated protein 3, KIF3A/3B, kinesin family member 3A/3B, MBP, maltose-binding protein, MDCK, Madin–Darby canine kidney, NLS, nuclear localization signal, NPC, nuclear pore complex, PY–NLS, proline-tyrosine NLS, TAP, Tris-acetate-phosphate, TIRFM, total internal reflection fluorescence microscopy, WB, Western blot

## Abstract

Kinesin is part of the microtubule-binding motor protein superfamily, which serves important roles in cell division and intraorganellar transport. The heterotrimeric kinesin-2, consisting of the heterodimeric motor subunits, kinesin family member 3A/3B (KIF3A/3B), and kinesin-associated protein 3 (KAP3), is highly conserved across species from the unicellular eukaryote *Chlamydomonas* to humans. It plays diverse roles in cargo transport including anterograde (base to tip) trafficking in cilia. However, the molecular determinants mediating trafficking of heterotrimeric kinesin-2 itself are poorly understood. It has been previously suggested that ciliary transport is analogous to nuclear transport mechanisms. Using *Chlamydomonas* and human telomerase reverse transcriptase-retinal pigment epithelial cell line, we show that RanGTP, a small GTPase that dictates nuclear transport, regulates ciliary trafficking of KAP3, a key component for functional kinesin-2. We found that the armadillo-repeat region 6 to 9 (ARM6–9) of KAP3, required for its nuclear translocation, is also necessary and sufficient for its targeting to the ciliary base. Given that KAP3 is essential for cilium formation and there are the emerging roles for RanGTP/importin β in ciliary protein targeting, we further investigated the effect of RanGTP in cilium formation and maintenance. We found that precise control of RanGTP levels, revealed by different Ran mutants, is crucial for cilium formation and maintenance. Most importantly, we were able to provide orthogonal support in an algal model system that segregates RanGTP regulation of ciliary protein trafficking from its nuclear roles. Our work provides important support for the model that nuclear import mechanisms have been co-opted for independent roles in ciliary import.

Cilia are microtubule-based protrusions with sensory and/or motile functions. In mammals, defects in assembly and maintenance of cilia result in a series of diseases called “ciliopathies” (1, 2, 3, 4). The assembly and maintenance of these organelles are dependent on anterograde and retrograde intraflagellar transport (IFT) (5). Anterograde IFT, which moves from the base of a cilium to the tip, is driven by kinesin-2 (6, 7), whereas retrograde IFT, which moves from the tip back to the base, is achieved by cytoplasmic dynein 1b (8, 9, 10). The kinesin-2 motor family is composed of a heterotrimeric kinesin family member 3A/3B (KIF3A/KIF3B)/kinesin-associated protein 3 (KAP3) motor and a homodimeric KIF17 motor (11). Unlike homodimeric KIF17, heterotrimeric kinesin-2 is highly conserved, and loss of function in any component of heterotrimeric kinesin-2 results in defective cilia in different organisms (12, 13, 14, 15). In addition to cilium formation, regulation and maintenance of cilium length is also dependent on the size and frequency of kinesin-2 trains recruited to and/or entering cilia (16, 17). In addition to its central role in IFT and ciliogenesis, heterotrimeric kinesin-2 has also been reported in other organelle transport events outside cilia. This includes anterograde transport from endoplasmic reticulum to the Golgi apparatus in *Xenopus* (18), retrograde transport from the Golgi to the endoplasmic reticulum in HeLa cells (19), and establishment of cell polarity during migration (20). Furthermore, heterotrimeric kinesin-2 is reported to play critical roles in mitosis (21, 22).

Cilia assemble in quiescent cells and disassemble in dividing cells (23). In quiescent cells, heterotrimeric kinesin-2 is localized in both cilia and basal body in ciliated cells. In dividing cells, when cells enter mitosis and cilia retract, the nonmotor subunit KAP is transported into the nucleus before nuclear membrane breaks down in cells of sea urchin blastulae (24). During cytokinesis, the motor protein KIF3B is localized in the midbody (21). Macromolecules cannot freely go into or out of the cilium or the nucleus because diffusion barriers exist at the ciliary base and the nuclear pore complex (NPC) (25, 26, 27, 28). The fundamental question is how this conserved heterotrimeric kinesin-2 complex traffics between different compartments (*i.e.*, the cytoplasm, nucleus, and cilium) for specific functions, and how these processes are regulated.

For nuclear transport from the cytoplasm, the NPC mediates active transport of proteins (29, 30, 31, 32). Larger proteins (>50 kDa) generally require RanGTP and specific importin transport receptors to cross the NPC (33, 34). Importins usually bind nuclear localization signal (NLS)–containing cargoes at relatively low RanGTP levels in the cytosol. This moves the complex through NPCs and releases cargo in the nucleus where RanGTP concentration is high (35, 36). In most cases, importin β1 binds to importin α, which interacts with a conventional NLS, to mediate substrate nuclear import (37). In contrast, importin β2 recognizes nontraditional proline–tyrosine NLS (PY–NLS) for nuclear import (38). In the nucleus, direct binding of RanGTP with importin β results in cargo release (39).

Compared with nucleocytoplasmic transport, the molecular mechanisms or sequence motifs controlling membrane or soluble protein trafficking into cilia are poorly understood. Previous studies reported that several cis-acting elements, including RVxP, VxPx, and Ax[S/A]xQ motifs, are important for mediating ciliary trafficking of membrane proteins (40, 41, 42, 43). Some have hypothesized that the cilium and nucleus have coevolved for signal integration because of the shared components and evolutionary origin (44, 45, 46). It was also proposed that there are some shared mechanisms between ciliary import and nuclear import (27, 28, 46, 47, 48). Several results indicated that the RanGTP/importin β/NLS import system is required for ciliary targeting of either membrane or soluble proteins, including importin β1 for Crumsb3 (49), RanGTP/importin β2 for KIF17 and Gli2 (46, 50), importin β2 for retinitis pigmentosa 2 (51), importin α1/α6/NLS for KIF17 (52), and importin β2/PY–NLS for Gli2/Gli3 (53). It was also reported that importin β2/RAB8 forms a ternary complex with ciliary localization sequences to direct membrane protein trafficking to cilia (54), suggesting that this process is independent of RanGTP and an NLS or PY–NLS. Despite these advancements in uncovering mechanisms for ciliary import, it remains unclear whether RanGTP regulates ciliary trafficking directly or by affecting nuclear import to result in defective ciliary trafficking. It is also unclear whether different cargoes depend on different importin β receptors for ciliary trafficking. Finally, prior work investigated determinants of ciliary entry for the homodimeric ciliary kinesin composed of KIF17 (46). However, the heterotrimeric kinesin-2 is the motor that dictates cilium assembly and maintenance (12, 13, 14, 15, 16, 17). Therefore, we focused on how ciliary trafficking of the heterotrimeric kinesin-2 is regulated by leveraging the unique advantages of the unicellular green alga *Chlamydomonas reinhardtii* as an excellent eukaryotic model to study ciliogenesis (5, 55).

Cilia of *Chlamydomonas* can be regenerated to full length in 2 h, are relatively homogenous, and do not need to be induced (56). This is in contrast to mammalian cells, which require ciliary induction and produce a heterogenous population of ciliated and nonciliated cells. The molecular mechanism of the nucleocytoplasmic trafficking is likely conserved between *Chlamydomonas* and humans (57). However, there are fewer constituent nucleoporins in the *Chlamydomonas* NPC compared with that of humans (58). By using both mammalian and *Chlamydomonas* cells, we have found that ciliary trafficking of KAP3 is regulated by RanGTP. We demonstrated that precise manipulation of RanGTP level is crucial for regulating cilium formation. Importantly, our data provide orthogonal support to existing data that RanGTP plays a direct role in incorporation of ciliary proteins that is independent of its nuclear roles. This further strengthens a model in which nuclear import mechanisms have been co-opted for direct ciliary import.

## Results

### The ciliary protein KAP3 can localize to the nucleus

**Figure 1:**
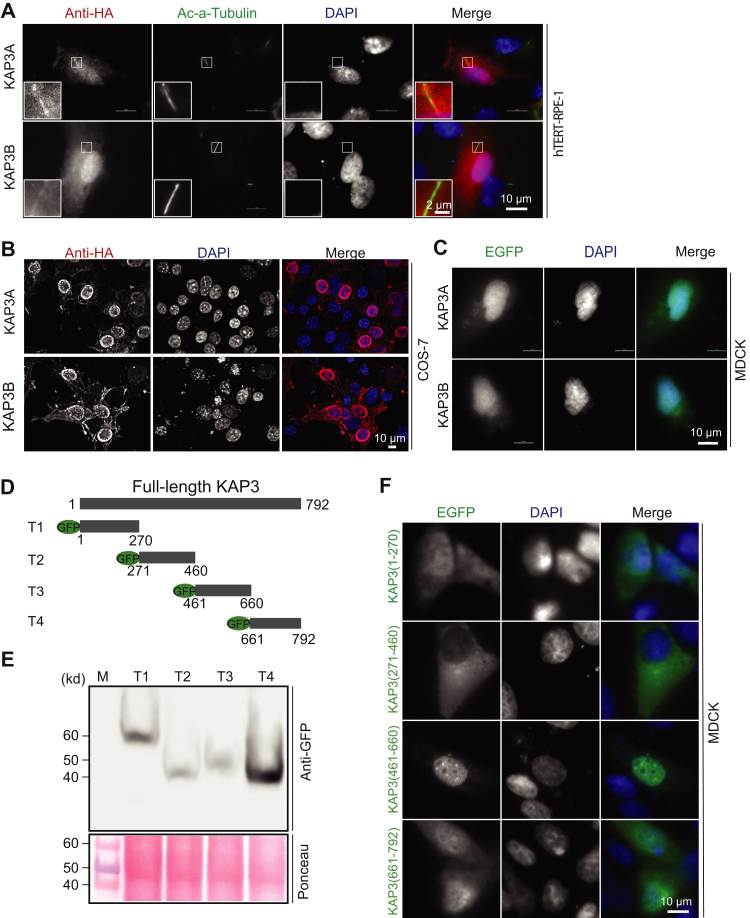
**Kinesin-associated protein KAP3 localizes in both the cilium and nucleus.***A*, HA-tagged KAP3A or KAP3B was transfected into hTERT-RPE cells. After 24 h transfection, the cilium was induced under 0.25% serum starvation for another 24 h. Cells were fixed with 4% paraformaldehyde and stained with rabbit anti-HA and mouse anti-ac-α-tubulin antibodies. The scale bar represents 10 μm; the scale bar for the zoomed inset panel is 2 μm. *B*, HA-tagged KAP3A or KAP3B was expressed in nonciliated COS-7 cells. After 24 h of transfection, cells were fixed with 4% PFA and stained with anti-HA antibody. Cell nuclei are pseudocolored *blue*, after staining with DAPI. The scale bar represents 10 μm. *C*, EGFP-tagged KAP3A or KAP3B was expressed in MDCK cells. *D*, schematic illustration of EGFP-tagged KAP3 truncated derivatives. The scale bar represents 10 μm. *E*, Western blotting analysis of the expression of EGFP-tagged KAP3 transiently transfected MDCK cells. *F*, mapping the domains required for nuclear localization of KAP3A in MDCK cells: a series of EGFP-tagged KAP3A truncations were transfected into MDCK cells. About 24 h after transfection, cells were fixed with 4% paraformaldehyde and stained with mouse anti-HA antibody and DAPI. The scale bar represents 10 μm. DAPI, 4ʹ,6-diamidino-2-phenylindole; EGFP, enhanced GFP; HA, hemagglutinin; hTERT–RPE-1, human telomerase reverse transcriptase–retinal pigment epithelial 1; KAP3, kinesin-associated protein 3; MDCK, Madin–Darby canine kidney.

The heterotrimeric kinesin-2 motor complex consists of the heterodimeric motor proteins KIF3A/3B and the adaptor protein KAP3. In contrast, the homodimeric kinesin-2 KIF17 motor does not need an adaptor protein to exert its function. Although it was suggested that KAP3 functions as a linker between KIF3A/3B and the specific cargoes to facilitate intracellular transport, the function of KAP3 is still not well characterized. To explore this, we first investigated the intracellular localization of KAP3 in ciliated and nonciliated cells. Hemagglutinin (HA)-tagged KAP3A and KAP3B (a short isoform of KAP3A) were transfected into human telomerase reverse transcriptase–retinal pigment epithelial (hTERT–RPE) cells. About 24 h after transfection, cilia were induced by serum starvation. As expected, both HA-tagged KAP3A and KAP3B colocalized with acetylated-α-tubulin, confirming that the intracellular localization of KAP3A and KAP3B is not affected by the small HA epitope and can be targeted to cilia (Fig. 1*A*). Surprisingly, we noticed that a significant amount of KAP3A and KAP3B was also distributed throughout the nucleus (Fig. 1*A*). We further examined the localization of KAP3A and KAP3B in other different cell types. As shown in Figure 1*B*, HA-tagged KAP3A and KAP3B are mainly localized in the nucleus of COS-7 cells, although a small amount is distributed in the cytoplasm. We also found that enhanced GFP (EGFP)-tagged KAP3A and KAP3B could localize in the nucleus of Madin–Darby canine kidney (MDCK) cells (Fig. 1*C*). Nuclear localization of KAP3A was consistent with that of EGFP-tagged KAP3A in a previous report (59).

To dissect critical regions within KAP3A responsible for its nuclear localization, four KAP3 truncations, named T1–T4, were generated and fused with EGFP (Fig. 1*D*). These truncations together cover the full-length KAP3, and the length of each truncation is around 200 amino acid residues (henceforth, KAP3 refers to the long isoform KAP3A). First, the expression of appropriately sized truncations in MDCK cells was detected by Western blot analysis (Fig. 1*E*). Second, the subcellular distributions of these KAP3 truncations in MDCK cells were analyzed *via* fluorescence microscopy. As shown in Figure 1*F*, the N-terminal fragment KAP3 (1–270) and the C-terminal fragment KAP3 (661–792) are distributed in both the cytoplasm and nucleus, and KAP3 (271–460) was exclusively distributed in the cytoplasm. In contrast, KAP3 (461–660), consisting of armadillo repeats (ARM) 6 to 9, were predominantly localized in the nucleus, which is similar to full-length KAP3. These data indicate that the region between amino acids 461 and 660 is crucial for nuclear localization of KAP3. Taken together, our data demonstrate that the ciliary protein KAP3 can localize to the nucleus under the control of ARM6–9.

### RanGTP, but not importin β2, mediates nuclear translocation of KAP3

**Figure 2:**
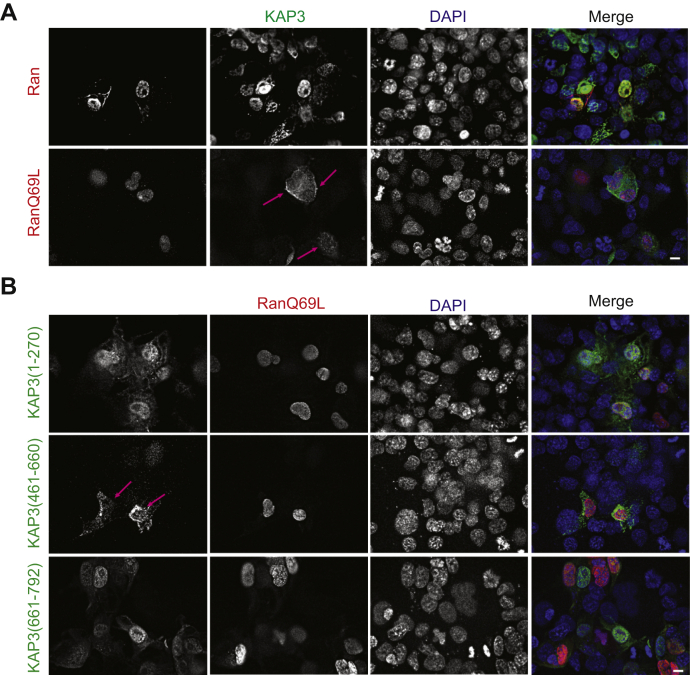
**RanGTP regulates nuclear translocation of KAP3.***A*, the dominant-negative form of Ran blocked nuclear localization of KAP3 in COS-7 cells. mCherry-tagged wildtype Ran or GTP-bound dominant mutant RanQ69L was cotransfected with enhanced GFP (EGFP)–KAP3 into COS-7 cells. After 24 h of transfection, cells were fixed with 4% paraformaldehyde and stained with anti-EGFP antibody and DAPI. The scale bar represents 10 μm. *B*, mapping the domains within KAP3 that is mediated by RanGTP mediated nuclear import. A series of EGFP-tagged KAP3 truncations were cotransfected with GTP-locked Ran mutant Q69L into COS-7 cells. After 24 h of transfection, cells were fixed with 4% paraformaldehyde and stained with anti-EGFP antibody and DAPI. The scale bar represents 10 μm. DAPI, 4ʹ,6-diamidino-2-phenylindole; KAP3, kinesin-associated protein 3.

As shown in Figure 1, KAP3 is distributed to the nucleus in different cells. To determine the molecular mechanism of KAP3 nuclear translocation, we tested a well-studied pathway for protein nuclear import, RanGTP-mediated nuclear import, which requires a high concentration of RanGTP in the nucleus for the disassembly of the imported complexes. To determine whether RanGTP drives nuclear import of KAP3, the dominant negative mutant RanQ69L, which cannot hydrolyze GTP, was used in this study. Ectopic expression of RanQ69L blocked nuclear localization of KAP3 in COS-7 cells, resulting in more cytoplasmic distribution of KAP3 relative to wildtype controls (Fig. 2*A*). These data suggest that nuclear translocation of KAP3 is mediated by a RanGTP-dependent nuclear import pathway.

We mapped the region responsible for nuclear localization of KAP3 and found that KAP3 (461–660) is required. If the region we mapped is correct, the nuclear localization of this truncated KAP3 (461–660) should be RanGTP dependent, which acts the same way as a full-length KAP3. To test this, we cotransfected KAP3 (461–660) with the dominant-negative Ran mutant RanQ69L. RanQ69L completely disrupted nuclear import of KAP3 (461–660) and resulted in the cytoplasmic localization of this truncation (Fig. 2*B*). These results suggest that RanGTP-dependent nuclear import of KAP3 is dependent on the 461 to 660 region of KAP3. In contrast, RanQ69L did not change the localization of other KAP3 truncations (Fig. 2*B*).

It was reported that the import receptor importin β2 plays critical roles in both nuclear import and ciliary import of ciliary proteins, like KIF17 and Gli2/Gli3 (46, 53). We further examined whether importin β2 is used for nuclear translocation of KAP3. The importin β2 inhibitory peptide M9M was used in these studies (60). Heterogenous nuclear ribonucleoprotein A1 (hnRNP A1) was used as a positive control for this assay. Expression of maltose-binding protein (MBP)–tagged M9M inhibitory peptide disrupted nuclear localization of hnRNP A1 (*white arrow*), which confirmed that hnRNP A1 uses transport receptor importin β2 for its nuclear translocation (Fig. S1*A*). Compared with the empty MBP control, MBP-tagged M9M did not block the nuclear translocation of KAP3 (Fig. S1*B*). These data suggest that nuclear import of KAP3 is independent of the importin β2 receptor.

### The armadillo-repeat region 6 to 9 (ARM6–9) alone is sufficient for ciliary base localization

**Figure 3:**
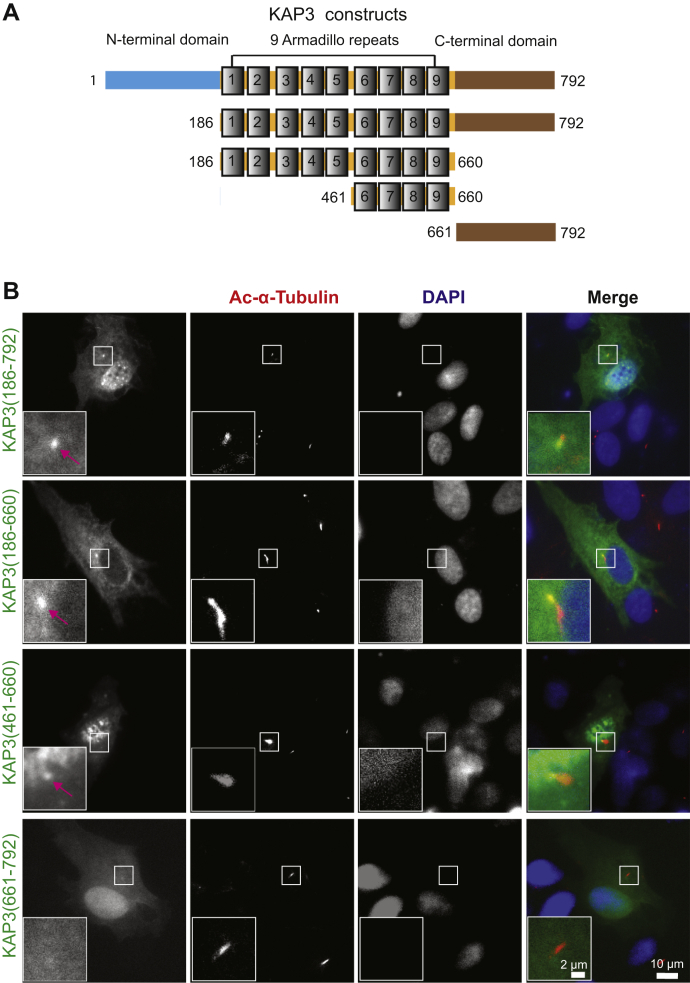
**Armadillo-repeat region 6 to 9 targets KAP3 to the ciliary base.***A*, schematic illustration of full-length human KAP3 and its truncated derivatives for analyzing ciliary targeting. *B*, ciliary base localization of KAP3 truncations in human telomerase reverse transcriptase–retinal pigment epithelial cells. These cells were transfected with various truncated constructs of KAP3. After 24 h of transfection, the cells were serum starved for cilium induction for another 24 h, fixed with 4% paraformaldehyde, and then costained with mouse anti-ac-α-tubulin (*red*) and rabbit anti-EGFP antibodies (*green*). Nuclei are stained with DAPI (*blue*). The scale bar represents 10 μm; the scale bar for the zoomed inset panel: 2 μm. DAPI, 4ʹ,6-diamidino-2-phenylindole; KAP3, kinesin-associated protein 3.

KAP3 can localize in both the cilium and nucleus. We dissected the regions required for nuclear translocation of KAP3. Next, we mapped the regions required for ciliary targeting of KAP3 in hTERT–RPE cells. As depicted in Figure 3*A*, full-length KAP3 contains three regions: a nonconserved N-terminal domain, nine armadillo repeats, and a C-terminal conserved domain (61, 62). Based on this, a series of truncations of KAP3 were generated, and intracellular localization of these truncations was examined after cilium induction. The truncation KAP3 (661–792) containing the C-terminal domain completely abolished localization to the cilium and ciliary base (Fig. 3*B*). In contrast, both the truncation KAP3(186–792) containing the C-terminal domain and nine armadillo repeats and the truncation KAP3(186–660) containing only the nine armadillo repeats showed intense signal at the ciliary base. These data suggest that the nine armadillo repeats are required for KAP3 targeting to the ciliary base. We further narrowed the region within the nine armadillo repeats and demonstrated that the truncation KAP3 (461–660) harboring ARM6-9 is sufficient for ciliary base targeting of KAP3 (Fig. 3*B*). It is noteworthy that this region is also required for RanGTP-mediated KAP3 nuclear trafficking (Fig. 1*F*).

### RanGTP regulates percent ciliation in hTERT–RPE cells

**Figure 4:**
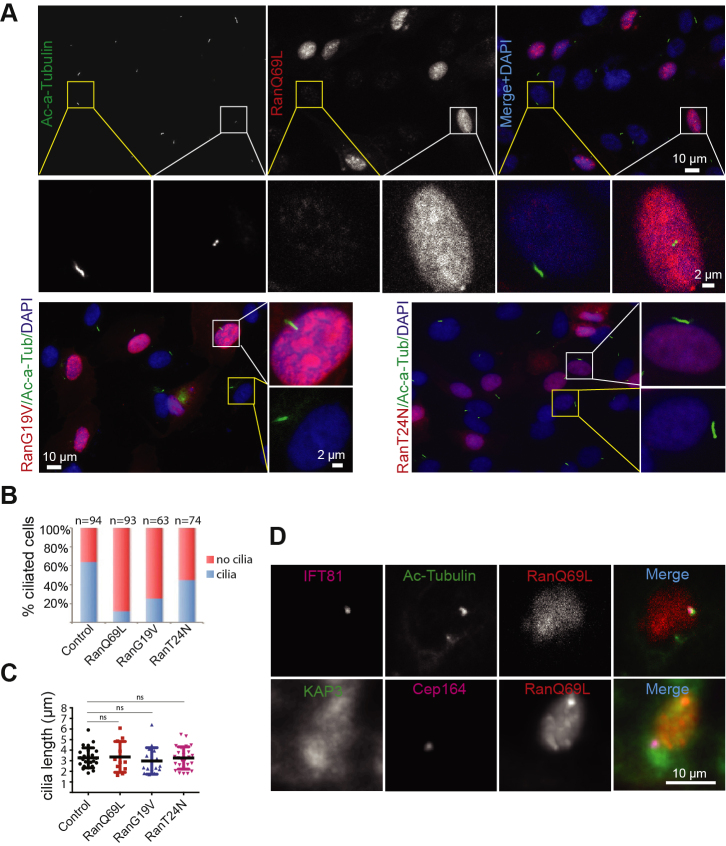
**Dominant-negative Ran mutants reduced percent ciliation in human telomerase reverse transcriptase–retinal pigment epithelial (hTERT–RPE) cells**. *A*, dominant-negative Ran mutants blocked cilia formation in hTERT–RPE cells. The plasmids expressing Ran mutant were transfected into hTERT–RPE cells. About 24 h after transfection, cilia were induced *via* serum starvation for another 24 h. The cells were fixed and stained for anti-ac-α-tubulin and DAPI. The scale bar represents 10 μm; the scale bar for the zoomed inset panel: 2 μm. *B*, hTERT–RPE cells expressing the dominant Ran mutant reduced the percentage of ciliated cells. *C*, quantification of cilia length. Data are presented as the mean ± S.D. The unpaired *t* test analysis was performed. *p* Values greater than 0.05 were not considered significantly different. The experiment was repeated three times. *D*, localization of IFT81 and KAP3 in ciliated hTERT–RPE cells expressing RanQ69L. Cilia were induced in hTERT–RPE cells expressing RanQ69L *via* serum starvation. The cells were fixed and costained with acetylated α-tubulin and IFT81 or KAP3 and Cep164. Nuclei are stained with DAPI. The scale bar represents 10 μm. DAPI, 4ʹ,6-diamidino-2-phenylindole; IFT, intraflagellar transport; KAP3, kinesin-associated protein 3.

Given the middle region of KAP3, 461 to 660, is required for both nuclear and cilium base targeting and nuclear targeting is RanGTP dependent, we wanted to investigate whether KAP3-dependent ciliogenesis and cilium length regulation (14, 63) were also RanGTP dependent. We were further interested in Ran-dependent ciliary phenotypes and KAP3 localization because of previously reported shared mechanisms between nuclear and ciliary import processes (27, 28, 46, 47, 48) and conflicting conclusions about the effect of RanGTP on ciliogenesis (46, 50, 64). Wildtype Ran and three well-characterized dominant-negative Ran mutants (RanQ69L, RanG19V, and RanT24N) were used in this study. First, we studied the intracellular localization of these proteins in hTERT–RPE cells in serum-starved conditions, which induce ciliogenesis. As shown in Figure S2, all the mutants are predominantly localized in the nucleus, which is similar to that of wildtype Ran. These data indicate that expression of these point mutants did not dramatically affect intracellular localization of Ran. To analyze the role of these mutants in cilium formation and length regulation in hTERT–RPE cells, wildtype and mutant Ran expression plasmids were transfected into hTERT–RPE cells. About 24 h post-transfection, low serum media were added for 24 h to induce cilium formation. Ectopic expression of either the GTP-locked mutants, RanQ69L/RanG19V, or the GDP-locked mutant, RanT24N, had no obvious effect on cilium length (Fig. 4, *A*–*C*). In contrast, cells transfected with these dominant-negative mutants could reduce ciliation percentage compared with the untransfected control cells (Fig. 4*B*). Furthermore, different Ran mutants had different effects on ciliation percentage. RanQ69L has higher affinity to GTP and resulted in a dramatic reduction in ciliation percentage compared with RanG19V, which has relatively low affinity for GTP (65) (Fig. 4*B*). These data indicate that the RanGTP level in hTERT–RPE cells is a determinant of initiation of cilium formation. Taken together, the ability to bind and hydrolyze GTP by Ran, revealed by different dominant Ran mutants, regulates its essential functions on the generation of cilia.

To determine why there is reduced cilium formation in RanQ69L-expressing RPE cells, we further investigated the localization of other important components including the IFT complex and kinesin-2. IFT81, a component of the IFT-B complex, is still localized in the ciliary base of hTERT–RPE cells expressing RanQ69L, suggesting that IFT-B targeting is not affected and unlikely to be the primary cause of defective cilium formation (Fig. 4*D*). In contrast, KAP3 did not localize to the ciliary base. These data suggest that, in addition to potential roles for RanGTP in ciliary entry, ciliary targeting of the heterotrimeric kinesin-2 is also RanGTP dependent.

### RanGTP regulates ciliary length and ciliary trafficking of KAP under steady-state conditions in *Chlamydomonas*

**Figure 5:**
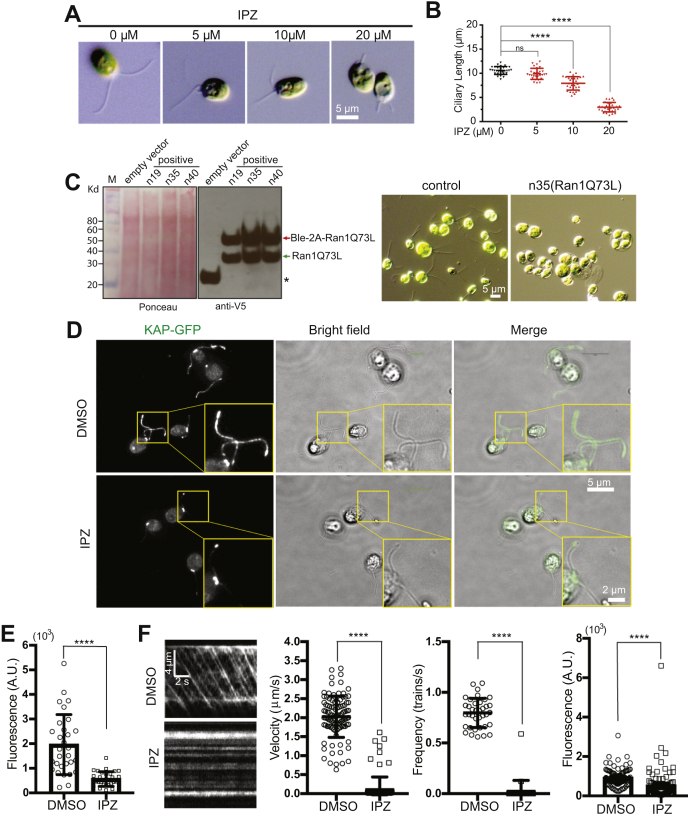
**Inhibition of RanGTP function shortens cilia length and blocks ciliary trafficking of KAP under steady-state conditions in *Chlamydomonas*.***A*, dose-dependent inhibition of cilia length by the small molecule inhibitor IPZ, which disrupts RanGTP interacting with importin β. CC-125 cells were treated with different concentrations of IPZ for 2 h, fixed with 1% glutaraldehyde, and imaged by differential interference contrast microscope at 40× magnification. The scale bar represents 5 μm. *B*, quantification of cilia length from (*A*). *C*, expression of the dominant-negative mutant Ran1Q73L, corresponding to human RanQ69L, exhibits either clumpy cells or shortened ciliary length in *Chlamydomonas*. The linearized expression plasmid pChlamy-4–Ran1Q73L was transformed into CC-125 cells and initially screened by colony PCR. The expression of Ran1Q73L was finally detected by Western blotting analysis *via* V5 antibody. Because of cleavage efficiency of 2A peptide in *Chlamydomonas*, Ran1Q73L existed in two forms: Ran1Q73L (correctly processed, *green arrow*) and Ble-2A–Ran1Q73L (fused with the selection marker, *red arrow*). Asterisks indicate that the negative control merely expresses zeocin resistance gene *Ble*. The representative images for the control cells and Ran1Q73L expressing cells are shown. The scale bar represents 5 μm. *D*, inhibiting Ran function by IPZ reduced ciliary localization of KAP. KAP–GFP reporter cells were treated with 20 μM IPZ for 2 h, fixed with 100% methanol, and mounted with prolong gold antifade mounting medium. Ciliary localization of KAP is dramatically reduced after IPZ treatment in *Chlamydomonas*. The scale bar represents 5 μm; the scale bar for the zoomed inset panel is 2 μm. *E*, quantification of KAP–GFP relative fluorescent intensity in cilia. Fluorescence measurements are relative to untreated cells. KAP–GFP cells were treated with 10 μM IPZ for 1 h, fixed with 100% methanol, and mounted with prolong gold antifade mounting medium. *F*, kymograph analysis indicates IPZ-blocked ciliary entry of KAP. KAP–GFP cells were treated with either DMSO or 10 μM IPZ for 1 h and then used for total internal reflection fluorescence microscopy to record the dynamic behavior of KAP. Velocity, frequency, and size of trains were quantified for each condition. Fluorescent measurements for train size is relative to the DMSO control. ∗∗∗∗*p* < 0.0001. DMSO, dimethyl sulfoxide; IPZ, importazole; KAP, kinesin-associated protein; ns, not significant.

To see if mechanisms of Ran-dependent ciliary targeting and entry are broadly conserved, we tested the effect of Ran manipulation on assembly and kinesin-2 motor targeting in *Chlamydomonas* cilia. The unicellular green alga *Chlamydomonas* is an excellent model to study ciliary length regulation and protein trafficking. In addition to the extensive body of literature on motor trafficking and ciliary assembly in this organism, the small G-protein Ran and key residues required for GTP hydrolysis are well conserved between humans and *Chlamydomonas* (Fig. S3). We therefore examined the role of Ran-like protein (Ran1), the ortholog of human Ran, on ciliary length regulation in wildtype *Chlamydomonas* CC-125 cells. To perturb RanGTP function, we used importazole (IPZ), a small molecular inhibitor that specifically blocks RanGTP–importin β1 interaction (66). Considering *Chlamydomonas* importin β is relatively divergent from mammalian importin β1, we first examined whether IPZ can disrupt RanGTP–importin β-mediated transport in *Chlamydomonas*. The transcriptional factor XAP5, which contains a classical NLS, is a putative substrate for RanGTP–importin β-mediated transport and mediates regulation of ciliary genes after severing and regeneration (57). We investigated the role of IPZ on transcriptional levels of XAP5-governed downstream genes (*Bbs8* and *Ift-144*) and nonregulated gene (*Ift-139*). *Lmln*, a gene for which expression is not dramatically changed at this time point during regeneration (67), was used as a loading control. IPZ treatment moderately inhibited the expression of *Bbs8* and *Ift-144* but not *Ift-139* during regeneration (Fig. S4). These data suggest that IPZ treatment could selectively inhibit cilia regrowth–associated gene expression in *Chlamydomonas*. This likely occurs through blocking nuclear import of NLS-containing substrates like XAP5, which is needed for normal ciliary gene expression during regeneration. We further investigated the role of IPZ on regulation of cilia length in *Chlamydomonas*. The results indicated that treating wildtype CC-125 cells with IPZ for 2 h shortens ciliary length in a dose-dependent manner (Fig. 5, *A*–*B*).

To exclude that the phenotype was caused by off-target effects of the inhibitor, the GTP-locked Ran1 mutant Ran1Q73L, corresponding to human RanQ69L, was transformed into *Chlamydomonas*. As shown in Figure 5*C*, there is strong Ran1Q73L expression as expected in *Chlamydomonas* (*green arrow*), despite a portion of expressed Ble-2A–Ran1Q73L fusion protein being incompletely processed because of the cleavage efficiency of 2A peptide in *Chlamydomonas* (*red arrow*). Compared with control cells with normal ciliary length and cell division, the cells expressing high levels of Ran1Q73L exhibit either clumpy cells or shortened ciliary length. One possible explanation for clumpy cells may be that there are no cilia to secrete ectosomes containing lytic enzyme to break the cell wall after cell division (68). These results demonstrated that RanGTP plays pivotal roles in ciliary length regulation in *Chlamydomonas*.

We showed that KAP3 could not be targeted to the ciliary base in hTERT–RPE cells constitutively expressing GTP-locked RanQ69L. It was reported that RanGTP regulates ciliary entry of the other motor KIF17, which is localized in the nucleus like KAP3 (46). Based on these data, we tested whether perturbing Ran function affected ciliary targeting or entry of KAP, the ortholog of human KAP3, in the *Chlamydomonas* KAP–GFP reporter strain CC-4296. KAP–GFP is distributed in the cilia in control cells treated with dimethyl sulfoxide (DMSO) (Fig. 5*D*). In contrast, ciliary localization of KAP–GFP was dramatically decreased in the cells treated with 20 μM IPZ for 2 h (Fig. 5*D*). These effects also hold at a lower IPZ dosage of 10 μM and incubation time of 1 h, and we used these milder conditions for further quantifications. As shown in Figure 5*E*, KAP–GFP fluorescence quantification indicates that KAP–GFP intensity is dramatically decreased in the cilia with IPZ treatment. These data suggest a specific effect of IPZ on ciliary localization of KAP–GFP. Taken together, our results indicate that ciliary localization of KAP is regulated by RanGTP in *Chlamydomonas*. To further investigate whether IPZ directly affects ciliary entry of KAP, we used real-time total internal reflection fluorescence microscopy (TIRFM) to study the dynamic behavior of KAP. Kymograph analysis shows that KAP enters into the cilium in untreated cells. In contrast, cells treated with 10 μM IPZ show a reduction in KAP–GFP train velocity, frequency, and size (Fig. 5*F*). These data indicate that RanGTP has a critical role in regulating ciliary entry and trafficking of KAP. We further examined KAP–GFP levels in isolated cilia under the same treatment (Fig. S5). Although an outlier in one trial resulted in no statistically significant difference overall, KAP–GFP levels were dramatically decreased in two of three trials. Combined with more sensitive and quantitative fluorescence intensity measurements in fixed cells (Fig. 5*E*), as well as the TIRFM quantification in live cells (Fig. 5*F*), the data in aggregate show a strong, consistent, and significant reduction in KAP–GFP within cilia on IPZ treatment.

### RanGTP directly regulates ciliary protein incorporation during cilia regeneration in *Chlamydomonas*

**Figure 6:**
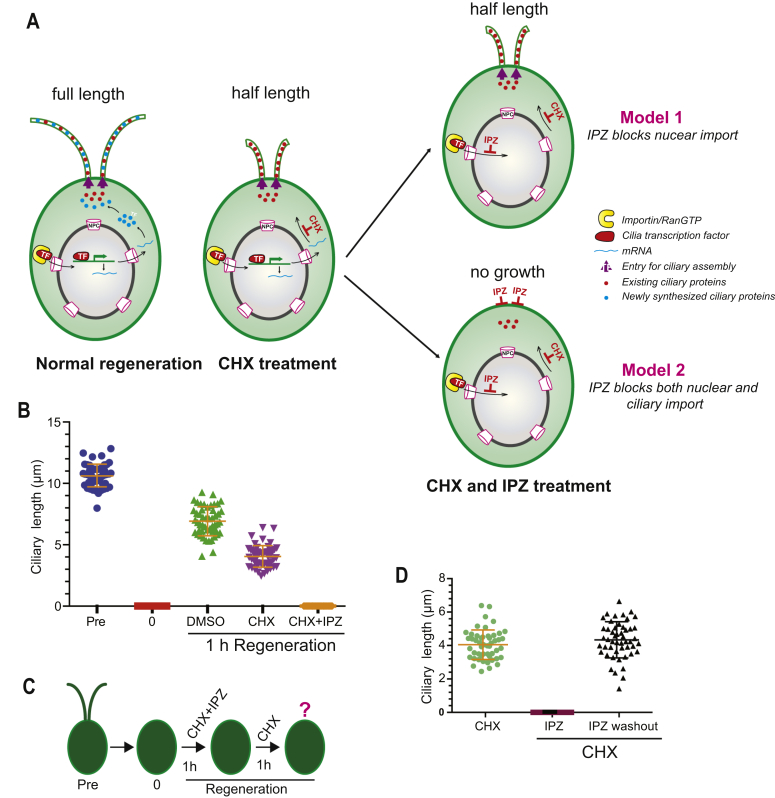
**RanGTP directly regulates ciliary protein incorporation in *Chlamydomonas* during ciliary regeneration.***A*, possible models of incorporation of existing ciliary protein into cilia after treatment with cycloheximide and importazole during ciliary regeneration. Inhibition of new protein synthesis by CHX results in half-length cilia. *B*, wildtype CC-125 cells were deciliated, and cilia were regenerated for 1 h in the presence of different small-molecule inhibitors: 10 μg/ml CHX, 10 μM IPZ, or the combination of 10 μg/ml CHX and 10 μM IPZ. Cells were fixed with 1% glutaraldehyde and imaged by a differential interference contrast microscope at 40× magnification. *C*, schematic representation of IPZ washout assay under the absence of newly synthesized protein. *D*, deciliated cells were treated with the combination of 10 μg/ml CHX and 10 μM IPZ for 60 min. Then IPZ, not CHX, was washed out, and cilia were regenerated for another 60 min. Cells were fixed with 1% glutaraldehyde, and cilia length was measured using ImageJ software. CHX, cycloheximide; DMSO, dimethyl sulfoxide; IPZ, importazole.

One advantage of the *Chlamydomonas* model system in this context is the significant available information about requirements for nuclear regulation of ciliary assembly. During ciliary regeneration after ciliary severing (deciliation), new ciliary proteins need to be synthesized and transported to assembly sites for incorporation into cilia (Fig. 6*A*). This process requires initiating gene expression, which would be dependent on nuclear import of specific transcription factors like XAP5 (57). Therefore, it is possible that RanGTP regulates cilium length by indirectly affecting nuclear import and ultimately affecting new transcription/ciliary protein synthesis. To tease apart nuclear and non-nuclear effects, we were able to use the small molecular inhibitor cycloheximide (CHX) to inhibit new protein synthesis during cilia regeneration. As shown in Figure 6*A*, in wildtype *Chlamydomonas* cells, this typically results in growth of cilia to half length (6 μm), which exhibits the ability of these cells to incorporate already-synthesized proteins to generate half-length cilia without the production of new proteins from the burst of transcription post-deciliation (56). As expected, when blocking new protein synthesis with CHX, the existing ciliary proteins can build short cilia (Fig. 6*B*). If RanGTP exclusively inhibits nuclear import, but not ciliary import, inhibition of Ran function should allow existing ciliary proteins to still incorporate and assemble cilia to half length (Fig. 6*A*; model 1). If inhibiting Ran function blocks both nuclear import and direct ciliary import, even the existing ciliary proteins should not incorporate and build cilia, resulting in bald cells (Fig. 6*A*; model 2). Our data fit model 2 and show that when the deciliated cells are treated with IPZ to inhibit Ran function (with CHX to block any new protein synthesis), there is no cilium formation during regeneration. This demonstrates that IPZ can directly block incorporation of the existing ciliary proteins into cilia for assembly (Fig. 6*B*). To confirm that the lack of ciliary growth was not because of cell toxicity and that IPZ only impacts the ability of existing proteins to enter cilia, we washed out IPZ but still continued CHX treatment to inhibit new protein synthesis. As shown in Figure 6, *C*–*D*, ciliary biogenesis is restored on IPZ washout. These data clearly show that under conditions where only existing ciliary proteins can either enter cilia or not, RanGTP has direct effects in regulating ciliary protein incorporation. We also released CHX inhibition to confirm that, regardless of the presence of new proteins, blocking Ran function can regulate incorporation of existing ciliary proteins expected to enter cilia on deciliation (Fig. S6, *A–B*). Our data indicate that IPZ can block incorporation of existing ciliary proteins in addition to any newly synthesized proteins. In these conditions, if IPZ blocked nuclear entry of transcription factors needed for the spike in ciliary proteins but did not directly affect ciliary entry of existing proteins, cilia would still reach half length from the already-synthesized ciliary protein pool. Ultimately, given the dual role of RanGTP in mediating ciliary import and nuclear import, it is important to segregate nuclear and direct ciliary effects of Ran perturbation. Here, we are able to show that in spite of its demonstrated roles in regulating nuclear protein import, RanGTP has direct roles in mediating ciliary protein incorporation for cilia formation.

## Discussion

Although most kinesin motors are localized in the cytoplasm, different conditions allow some kinesin motors to transport into the nucleus, including KAP3, KIF4, KIF17, and KIF17B (24, 46, 69, 70). KAP3 and KIF4 can redistribute to the nucleus during mitosis (24, 69). During mouse spermatid development, KIF17B shuttles from nucleus to cytoplasm (70). We observed that both isoforms of the heterotrimeric kinesin-2 accessory subunit, KAP3A and KAP3B, are localized in the nucleus, and their nuclear localization is RanGTP dependent. Considering KIF17B can function as a transcriptional regulator (70), it is possible that KAP3 participates in regulation of gene expression in the nucleus. Besides the kinesin motors, many cilia-associated proteins can localize to the nucleus (71). The exchange of components between the ciliary and nuclear compartments is also thought to be mediated by membrane-less organelles (44). However, the nuclear roles and origin of cilia-associated proteins need to be further investigated.

It was reported that the armadillo repeats of KAP3 are responsible for binding to motor subunits KIF3A/3B, and the C-terminal conserved domain is responsible for specific cargo binding (22, 61, 72, 73). Our data show that the ARM6–9 are required for KAP3 targeting to the ciliary base, probably mediated by RanGTP. It is possible that the heterodimeric KIF3A/3B and RanGTP collaboratively regulate KAP3 targeting to the ciliary base. It is also noteworthy that cells expressing the truncated KAP3 (186–660) with only the armadillo-repeat domain have normal cilia length, whereas cells expressing the truncated KAP3 (186–792) with both the armadillo repeats and cargo-binding domains have no cilia. Given that loss of the cargo-binding domain dramatically decreases KAP3 binding to KIF3A/3B (22), our data suggest that the dominant-negative function of KAP3 truncations is dependent on their ability to bind to the KIF3A/KIF3B motor subunits.

Several lines of evidence suggest that RanGTP is involved in ciliary protein trafficking (46, 51, 64, 74). RanGTP was reported to regulate ciliary entry of the homodimeric motor KIF17 and retinitis pigmentosa 2 (46, 51). RanGTP was also reported to facilitate ciliary export of huntingtin (74). However, the role of RanGTP on cilium formation was ambiguous. Two groups demonstrated that RanGTP has no effect on ciliary biogenesis (46, 50), whereas another group showed that manipulation of RanGTP concentration *via* RanBP1 knockdown could drive cilia formation (64). The difference between these observations for the role of RanGTP on cilia formation may be due to different cell lines, different strategies for inhibiting RanGTP function, or the timing and levels of RanGTP inhibition relative to cilia induction. Our results indicate that different dominant-negative forms of Ran have different effects on cilia formation, although these mutants have no effect on regulating cilium length. Furthermore, GTP-locked mutant RanQ69L more dramatically affects percent ciliation than that of RanG19V. The difference between RanQ69L and RanG19V is that RanQ69L has much higher affinity for GTP than RanG19V, thus RanQ69L-expressing cells have less free RanGTP than RanG19V-expressing cells. Taken together, our results suggest that precise control of intracellular free RanGTP is critical for regulating cilium formation.

In addition to RanGTP, the importin transport receptors also participate in ciliary protein trafficking (46, 49, 50, 53, 54). There is also some disagreement about which importin is used for ciliary protein trafficking. Importin β1 was responsible for transmembrane protein Crumbs3 ciliary trafficking (49), and importin β2 was identified as the transport receptor for ciliary targeting of either transmembrane or soluble proteins like KIF17, Gli2, and Gli3 (46, 50, 51, 53, 54). However, additional data have shown that importin α1 and α6, but not importin β2, are responsible for ciliary targeting of soluble KIF17 (52). In general, importin β1, alone or in cooperation with importin α, transports substrates with a conventional NLS (75), whereas importin β2 transports substrates that contain the nonconventional PY–NLS (38). Consistent with this, ciliary targeting of the transcriptional factor Gli2/Gli3, which uses transport receptor importin β2, relies on its PY–NLS motif. PY–NLS mutations also result in the loss of Gli2/Gli3 ciliary targeting (53). It is reported that the NLS-like sequence in the C-terminal region of KIF17 is required for its ciliary targeting (46). This NLS-like sequence was further confirmed as a classical monopartite NLS (52). However, we noticed that proline-lysine, the PY variant, is located in the immediate downstream region of this NLS. It is worth investigating whether or not this C-terminal NLS of KIF17 is a PY–NLS and which importin is used for KIF17 ciliary trafficking. Recently, a ternary complex consisting of importin β2, small GTPase RAB8, and ciliary targeting signals was reported to guide transmembrane protein trafficking to the cilium (54). These data suggest that spatial structure of the ternary complex, but not specific ciliary targeting sequences, is required for ciliary targeting of membrane proteins. This highlights that the detailed working model for how importin mediates ciliary import needs to be further clarified.

There are increasing data that nuclear import and ciliary import share similar mechanisms, at least in part (25, 27, 28, 46, 47, 48). First, both the NPC and the ciliary pore complex form a diffusion barrier (25, 28). Second, the RanGTP/importin transport system is also used for ciliary protein trafficking (46, 49, 51, 53). Third, some nucleoporins also localize in the ciliary base to regulate barrier diffusion ability (28, 46, 47). Our data have shown that RanGTP can regulate cilium formation and ciliary trafficking of KAP3. One remaining critical question is whether RanGTP has direct effects in modulating ciliary protein transport. One possibility is that the effect of RanGTP is an indirect result of inhibiting nuclear import of proteins, like transcription factors, which are required for ciliary formation. By using the unicellular green alga *Chlamydomonas* as a model organism, we clearly demonstrated that RanGTP function directly regulates ciliary incorporation of the existing pool of already-synthesized ciliary proteins, which is not dependent on new transcription. In addition, the dominant-negative mutant RanQ69L blocked ciliary trafficking of KAP3. Given that KAP is required for localization of KIF3A/3B to the assembly sites (63), RanGTP may control cilia formation by directly regulating ciliary targeting of the heterotrimeric kinesin-2 motor. This will in turn affect ciliary assembly and length maintenance because of the importance of ciliary recruitment and entry of the kinesin-2 motor KIF3A/3B/KAP in these processes (16, 17). Further work will determine if this is a generalized mechanism for ciliary protein import and will identify additional RanGTP-regulated ciliary proteins (cargoes) required for cilium assembly, length control, and function.

## Experimental procedures

### Compounds

DMSO, IPZ (#SML0341), and CHX (C1988) were purchased from Sigma-Aldrich. Indicated concentrations and specific incubation times are used in this study.

### DNA constructs

Plasmids for HA-tagged human KAP3A and KAP3B were kindly from Dr Benjamin Allen (University of Michigan). Plasmids for wildtype Ran and point mutants RanG19V and RanT24N are a generous gift from Dr Kristen Verhey (University of Michigan). Plasmids expressing MBP and M9M were from Dr Yuh Min Chook (University of Texas Southwestern Medical Center). Plasmids pmCherry-C1–RanQ69L (#30309) were obtained from Addgene under the material transfer agreement. GeneArt *Chlamydomonas* protein expression vector pChlamy_4 was from Thermo Fisher Scientific. Recombinant plasmids pChlamy_4_Ran1Q73L, EGFP or HA-tagged KAP3 truncations were generated by ligation-independent cloning as described before (76) and sequenced in full.

### *Chlamydomonas* strains, mammalian cells, and antibodies

Wildtype and KAP–GFP reporter strains were obtained from the *Chlamydomonas* resource center (CC-125 mt^+^ and CC-4296). Strains were grown in liquid Tris-acetate-phosphate (TAP) liquid medium for 18 to 24 h before experimentation. Mammalian COS-7 and MDCK cells were cultured in Dulbecco's modified Eagle's medium (DMEM; Invitrogen) supplemented with 10% fetal bovine serum (FBS; Invitrogen). Human TERT–RPE cells were cultured in DMEM + F12 (1:1) (Invitrogen) containing 10% FBS. Antibodies used in this study are as follows (IF and WB are short for immunofluorescence and Western blot, respectively): mouse antiacetylated α-tubulin (#T6793; 1:500 for IF) was from Sigma-Aldrich (St Louis, MO); rabbit anti-Cep164 (#22227-1-AP; 1:50 for IF) and rabbit anti-IFT81 (#11744-1-AP; 1:50 for IF) were from Proteintech; mouse anti-KAP3A (#610637; 1:20 for IF) was from BD Transduction Laboratories; mouse anti-Myc (AB_390912; 1:100 for IF) was from Roche; mouse anti-hnRNP A1 antibody (sc-32301; 1:50 for IF) was from Santa Cruz Biotechnology; rabbit anti-myc (#5625; 1:500 for IF), rabbit anti-V5 (#13202; 1:1000 for WB), rabbit anti-HA (#3724; 1:100 for IF), and rabbit anti-GFP (#2956; 1:100 for IF and 1:1000 for WB, respectively) were from Cell Signaling Technology. Goat anti-rabbit horse radish peroxidase (#G21234; 1:5000) was from Invitrogen.

### Cell culture and transfection

COS-7, MDCK, and hTERT–RPE cells were maintained in a humidified atmosphere at 37 °C and 5% carbon dioxide. Cells for transfection were seeded in an 8-well chamber slide (Lab-Tek) with 0.4 ml culture medium per well. After overnight growth, the cells became 70% to 80% confluent and were transfected with the corresponding plasmids using the transfection reagent FuGENE 6 (Roche) according to the manufacturer's instructions. In normal condition, COS-7, MDCK, and hTERT–RPE cells are fixed with 4% paraformaldehyde for the intracellular localization assay 24 h post-transfection. In serum-starved conditions for cilium induction, hTERT–RPE cells were cultured in complete medium for 24 h post-transfection, then followed to culture in DMEM + F12 (1:1) with 0.25% FBS for another 24 h.

### *Chlamydomonas* transformation

Electroporation transformations were performed on *Chlamydomonas* with the electroporator NEPA (Nepa Gene, Japan). Transformations were performed following the published protocol with some modifications (77). The typical 4 days were necessary to perform the transformation. *Day 1*—the cells were grown in 5 ml TAP liquid medium for overnight culture. *Day 2*—precultured cells from day 1 were transferred into a new 50 ml TAP medium in a 250 ml flask with a final absorbance at 730 nm of 0.1 (usually 1–3 ml precultures added) for overnight culture with 120 rpm/25 °C. *Day 3*—cells were harvested by centrifugation when the cell density reached an absorbance at 730 nm of 0.3 to 0.4 and washed by GeneArt MAX Efficiency Transformation Reagent (Invitrogen) three times and resuspended in 250 μl TAP medium containing 40 mM sucrose. About 1.6 μg linearized DNA (pChlmay_4_Ran1Q73L) was mixed with 160 μl of the cell suspension for electroporation. After electroporation, the cells were transferred into 10 ml TAP plus 40 mM sucrose for overnight culture in dim light. *Day 4*—cells were collected and plated onto 1.5% TAP-agar plate with 10 μg/ml zeocin for growth. The colonies were visible 5 to 7 days later.

### RNA extraction and RT-PCR

CC-125 cells were treated with 10 μM IPZ for 60 min during cilia regeneration (DMSO was used as a control). Cells were harvested, and total RNA was isolated with GeneElute Total RNA purification Kit (Sigma-Aldrich). On-column PureLink DNase I (Thermo Fisher Scientific) digestion to remove genomic DNA contamination was then performed. For reverse transcription, 1 μg purified total RNA was used for first-strand cDNA synthesis with SuperScript III First-Strand Synthesis kit (Thermo Fisher Scientific). Generation of gene-specific primers for amplification of *Bbs8*, *Ift-139*, *Ift-144* was described previously (57). A pair of primers (forward: 5ʹ-CCAACGGCACCAGCTC-3ʹ and reverse: 5ʹ-AGCTCCGCCCAGAAGG-3ʹ) was used for amplification of the expression control Lmln. PrimeSTAR HS DNA Polymerase (TaKaRa) was used for the PCR assay, and the amplification conditions were as follows: 94 °C for 30 s, followed by 30 cycles of 98 °C for 10 s, 55 °C for 5 s, and 72 °C for 60 s.

### Immunofluorescence staining

Cells were washed with cold PBS twice and then fixed with 4% paraformaldehyde in Hepes (pH 7.4) for 15 min at room temperature. Cells were washed three times with cold PBS and then incubated with 0.1% Triton X-100 in PBS (pH 7.4) for 10 min. Permeabilized cells were washed with PBS three times and then incubated in PBS with 10% normal goat serum and 1% bovine serum albumin (BSA) for 1 h at room temperature to block nonspecific binding of the antibodies. Cells were incubated with diluted primary antibody in PBS with 1% BSA overnight at 4 °C. After three washes with PBS, cells were incubated with the secondary antibody in PBS with 1% BSA for 1 h at room temperature in the dark. After three washes with PBS, cells were mounted with ProLong Antifade mounting medium with or without 4ʹ,6-diamidino-2-phenylindole and kept at 4 °C in the dark for further imaging.

### KAP–GFP quantification

Cells expressing KAP–GFP were treated with DMSO or 10 μM IPZ for 1 h and then fixed immediately in 100% methanol twice for 5 min. Fixed cells were imaged on Nikon Ti-E microscope at 100×. A series of Z-stacks were acquired with 0.2 μm z-step. Image deconvolution was performed using NIS-Elements software. KAP–GFP levels at basal bodies and cilia were quantitatively analyzed with ImageJ software using background-subtracted total fluorescence of the cilia calculated by subtracting the product of the area of the cilia times the background from the integrated density. KAP–GFP levels at the cilium were averaged by cilium length.

### TIRFM and quantification

Samples were prepared as follows: KAP–GFP reporter cells (CC-4296) were cultured in TAP liquid medium for 18 h and then centrifuged at 1000 rpm for 2 min. The 3 μl cell pellets were resuspended in 200 μl TAP liquid medium containing either DMSO or 50 μM IPZ. Coverslips of 24 × 50 mm no. 1.5 were treated with 0.1% polylysine for 10 min, dipped into water, and allowed to air-dry. Petroleum jelly was used to draw a circle around polylysine-coated regions. Cells of 10 μl were placed inside the hydrophobic circle and allowed to settle for 3 min before imaging. KAP–GFP cells were imaged on a Nikon Ti-E microscope with a 100× 1.49 numerical aperture oil objective. Images were obtained at 9.97 fps with 0.16 μm per pixel by using an Andor DU897 electron-multiplying charge-coupled device camera. Kymographs of flagella were built and analyzed in ImageJ using KymographBuilder. For velocity assay, the angle of the trains with respect to the basal body was recorded. We took the tangent of the angle in radians and converted this to distance over time using the recorded frames per second. To calculate frequency, the number of trains present were counted and divided by the total number of pixels converted to time using the recorded frames per second.

### Cilia isolation and immunoblot

Cilia were isolated using 25 mM dibucaine from *Chlamydomonas* according to the previous report with some modifications (78). Briefly, cells were grown to an absorbance at 730 nm of ∼1.6. After treatment with either 0.5% DMSO or 10 μM IPZ for 1 h, cells were centrifuged at 1100*g* for 3 min and washed with 10 mM Hepes (pH 7.4) twice before resuspending in 10 mM Hepes (pH 7.4), 5 mM magnesium sulphate, 1 mM DTT, 4% (w/v) sucrose (HMDS), and removing flagella with 25 mM dibucaine. After dibucaine treatment, we added HMDS with 0.5 mM EGTA and then centrifuged cells at 1100*g* for 3 min. The supernatant was underlayed with HMDS containing 25% sucrose. This suspension was centrifuged at 2400*g* for 10 min with the brake set to zero. We divided the sucrose gradient into 2 ml fractions and then centrifuged these fractions at 21,000*g* for 30 min at 4 °C to concentrate cilia. Cilia were resuspended in lysis buffer (5% glycerol, 1% Nonidet P-40 substitute, 5% TAP, 1 mM DTT, and 1× protease inhibitor [Thermofisher; #1861280]). A Bradford assay was used to determine protein concentration. Total ciliary proteins were boiled at 70 °C in 1× loading buffer (Thermofisher; #NP0008) and 50 mM DTT for 10 min and then run on SDS-PAGE. Proteins were transferred to nitrocellulose membrane and probed with anti-GFP primary antibody, goat anti-rabbit horse radish peroxidase secondary antibody and pico chemiluminescent substrate (Thermofisher; 3458) for KAP–GFP and imaged on the Bio-Rad ChemiDoc Imaging System. Intensity of anti-GFP bands was normalized to total protein visualized with amido black staining and imaged on the Bio-Rad ChemiDoc.

### Ciliary regeneration

*Chlamydomonas* cells were deciliated by pH shock as described before (79), and ciliary regeneration was induced in normal TAP liquid medium. After deciliation, cells were immediately treated with 10 μg/ml CHX and/or 10 μM IPZ for 60 min. For IPZ washout experiments, treated cells were washed three times with TAP medium and cultured in fresh TAP liquid medium or with 10 μg/ml CHX. Cells were fixed with 1% glutaraldehyde for 15 min at room temperature, and cilia lengths were measured using the line segment tool in ImageJ.

### Statistical analyses

All data are reported as mean values ± S.D. Graphs and associated statistical analyses were performed with Prism 6.0C (GraphPad Software, La Jolla, CA). The unpaired Student's *t* test was used to assess statistical significance of two groups. A value of *p* < 0.05 was considered statistically significant.

## Data availability

All representative data are contained within the article.

## Conflict of interest

The authors declare that they have no conflicts of interest with the contents of this article.
