# Supplementary material for: Separable roles for RanGTP in nuclear and ciliary trafficking of a kinesin-2 subunit
Source: J Biol Chem. 2020 Dec 3;296:100117. doi: 10.1074/jbc.RA119.010936 (PMC7948393; doi:10.1074/jbc.RA119.010936)
Supplement: Figures S1 to S6 [file mmc1.pdf]

Supporting information

Separable roles for RanGTP in nuclear and ciliary trafficking of a kinesin-2 subunit

Shengping Huang [2*], **Larissa L. Dougherty**[1,3] **Prachee Avasthi** [1,2,3*]

[1] Anatomy and Cell Biology, University of Kansas Medical Center, Kansas City, KS;
[2] Ophthalmology, University of Kansas Medical Center, Kansas City, KS.
[3] Biochemistry and Cell Biology, Geisel School of Medicine at Dartmouth College, Hanover, New Hampshire
*Addresses for correspondence: Shengping Huang (E-mail address: sphalan@gmail.com); Prachee Avasthi (Email address: Prachee.Avasthi@darthmouth.edu)

Running title: RanGTP in nuclear and ciliary trafficking

Key words: RanGTP, Kinesin-2, KAP3, nuclear import, ciliary targeting, *Chlamydomonas*

Supplementary figure legends

Figure S1. **Nuclear translocation of KAP3 is transport receptor importin β2 independent. A.** Importin β2 mediates nuclear translocation of hnRNP A1. The plasmid myc-MBP-M9M expressing myc-MBP fused inhibitory peptide M9M of importin β2, or its control merely expressing myc-MBP, were transfected into COS-7 cells. 24 h post transfection, cells were fixed with 4% PFA and co-stained with rabbit anti-myc (Red) and mouse anti-hnRNP A1 antibodies (green). Nuclei were stained with DAPI (blue). Scale bar: 10 µm. **B.** Importin β2 is not required for nuclear localization of KAP3. Importin β2 mediates nuclear translocation of hnRNP A1. The plasmid myc-MBP-M9M or its control were co-transfected with EGFP-KAP3 into COS-7 cells. 24 h post transfection, cells were fixed with 4% PFA and co-stained with mouse anti-myc (Red) and rabbit anti-EGFP antibodies (green). Nuclei are stained with DAPI (blue). Scale bar: 10 µm.

Figure S2. Subcellular localization of wild-type Ran and its dominant negative mutants in serum starved hTERT-RPE cells. Plasmids expressing wild-type Ran or its point mutants (RanQ69L, RanG19V and RanT24N) were transfected into hTERT-RPE cells. 24 h post transfection, the cells were serum starved for another 24 h and then fixed with 4% PFA. The intracellular localization of these proteins was visualized via immunofluorescence staining. Scale bar: 10 µm.

Figure S3. Alignment of human Ran with *Chlamydomonas* Ran-like small GTPase (Ran1). Human Ran (NCBI reference sequence: NP_006316.1) and *Chlamydomonas* Ran1 (Phytozome reference sequence: cre03.g191050.t1.2) were aligned using the Clustal Omega software. Conserved residues are indicated (asterisks indicate fully conserved residues; colons indicate residues with similar properties; periods indicate residues with weakly similar properties). The key residues required for the GTP or GDP bound state of Ran are marked in red.

Figure S4. The small molecular inhibitor IPZ selectively down-regulates the expression of cilia regrowth-associated genes in *Chlamydomonas*. A. CC-125 cells were treated with 10 µM IPZ or DMSO for 60 min during cilium regeneration. Total RNA was extracted and the first strand cDNA was synthesized**.** The transcriptional levels of different genes (*Bbs8, Ift-139, Ift-144*)

were analyzed by RT-PCR. *Lmln* was used as a housekeeping control. Three biological repeats were performed in the experiment. **B.** Quantification of the intensity of RT-PCR products. The relative values of each gene were divided by the housekeeping control and then normalized with the DMSO treated group.

Figure S5. Immunoblot analysis of KAP-GFP protein levels in isolated *Chlamydomonas* cilia with IPZ treatment. KAP-GFP cells were treated with 10 μM IPZ or DMSO for 1 hour. Total flagellar proteins were isolated and run on a 10% SDS-PAGE gel. Blots were then probed with anti-GFP antibody and imaged on the Bio-Rad ChemiDoc Imaging System. The relative Intensities of KAP-GFP were normalized to total cilia proteins visualized with amido black staining. “ns” means “not significant”.

Figure S6. RanGTP regulates ciliary protein incorporation in *Chlamydomonas* regardless of the presence of newly synthesized proteins. **A.** Possible models for RanGTP regulating ciliary protein incorporation in the presence of newly synthesized proteins. **B.** Deciliated cells were treated with 10 μM IPZ for 60 min. IPZ was washed out and cilia were regenerated for another 60 min. Cells were fixed with 1% glutaraldehyde and cilia length was measured using ImageJ software.

Figure S1

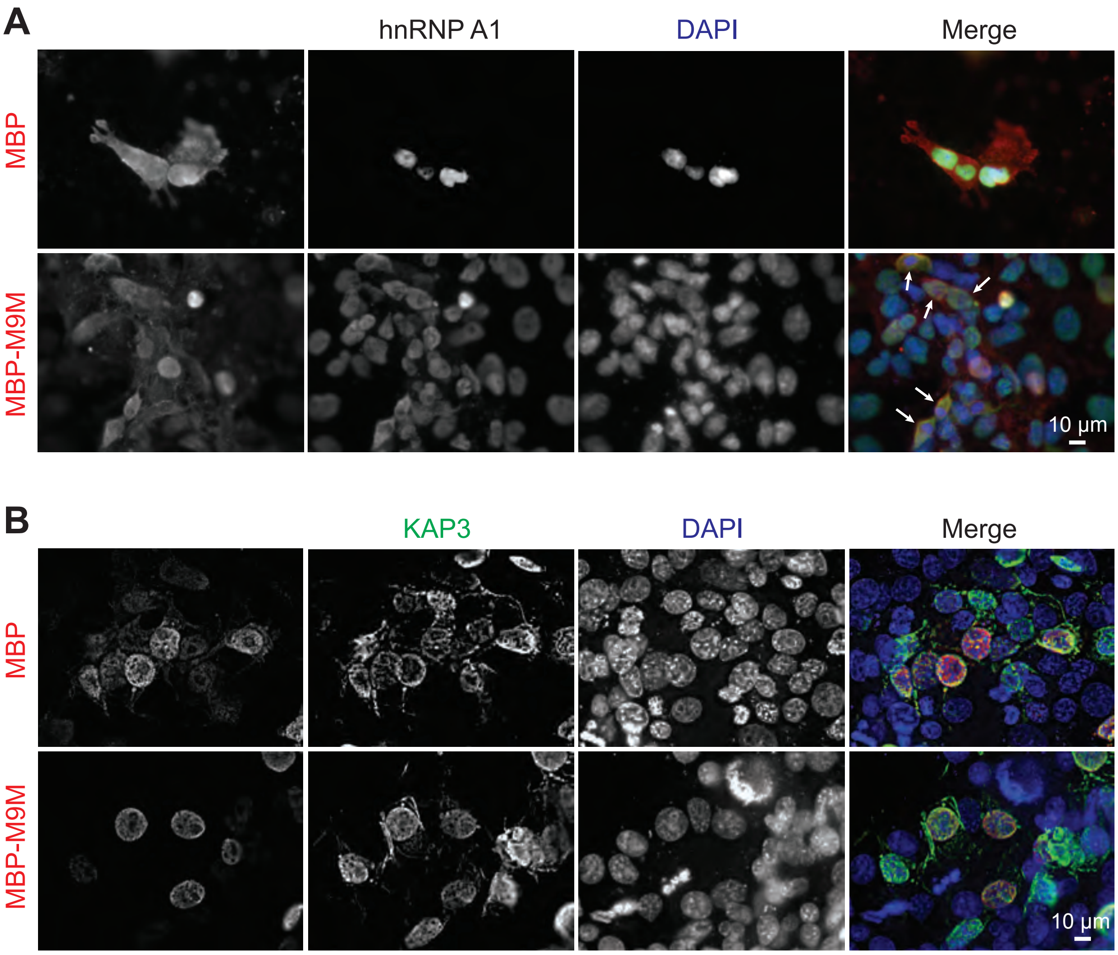

Figure S1. Nuclear translocation of KAP3 is transport receptor importin β2 independent

Figure S2

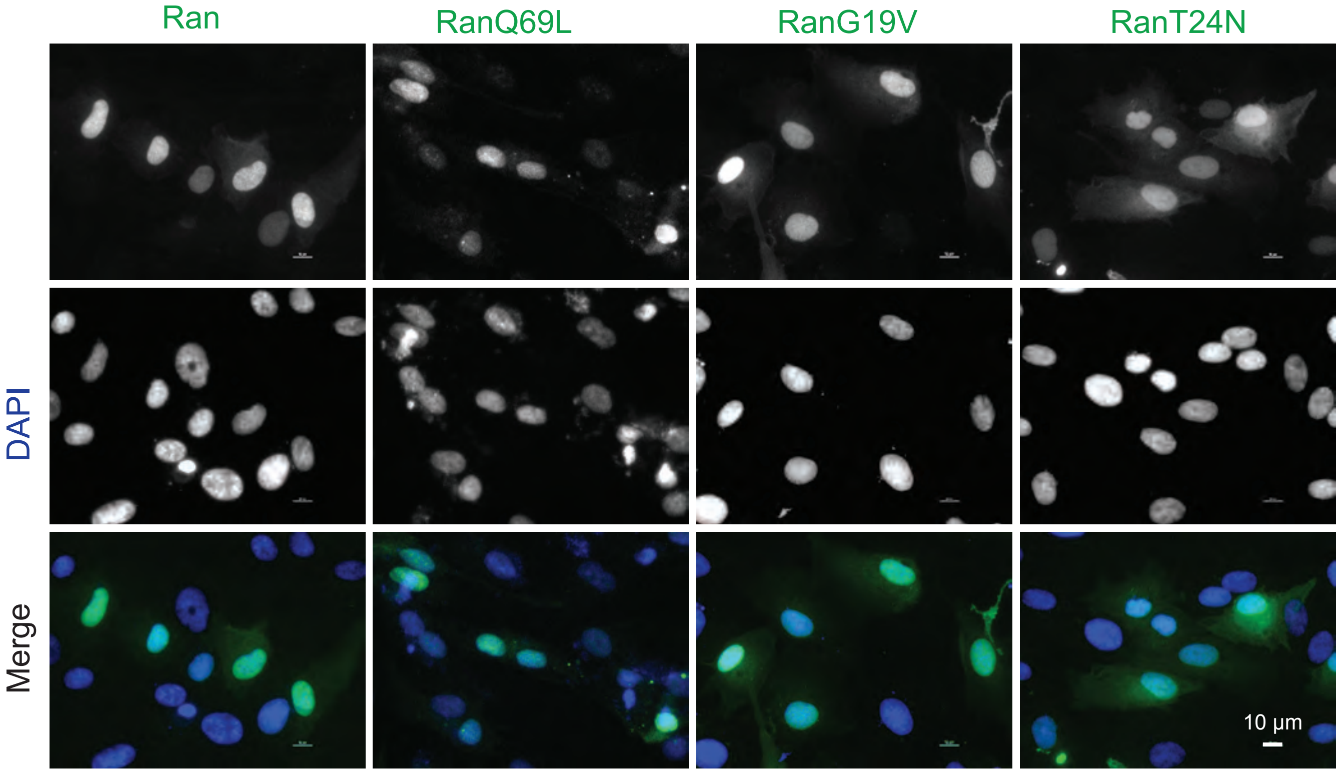

Figure S2. Subcellular localization of wild-type Ran and its dominant negative mutants in serum starved hTERT-RPE cells

Figure S3

```
human Ran      MA----AQGEPQVQFKLVLVGDGGTGKTTFVKRHLTGEFEKKYVATLGVEVHPLVFHTNR 56
Chlamy Ran1    MALPGQTTPEGVPAFKLVLVGDGGTGKTTFVKRHITGEFEKKYEPTIGVEVRPLDFTTNR 60
               **    :  *    ********************:********  *:****:** * ***

human Ran      GPIKFNVWDTAGQEKFGGLRDGYYIQAQCAIIMFDVTSRVTYKNVPNWHRDLVRVCENIP 116
Chlamy Ran1    GKIRFYCWDTAGQEKFGGLRDGYYIHGQCAIIMFDVTSRLTYKNVPTWHRDLCRVCENIP 120
               * *:*  ******************:.************:******.***** *******

human Ran      IVLCGNKVDIKDRKVKAKSIVFHRKKNLQYYDISAKSNYNFEKPFLWLARKLIGDPNLEF 176
Chlamy Ran1    IVLCGNKVDVKNRQVKPKQVTFHRKKNLQYYEISAKSNYNYEKPFLYLARKLTGDPHLSF 180
               *********:*:*:** *.:.**********:********:*****:***** ***:*.*

human Ran      VAMPALAPPEVVMDPALAAQYEHDLEVAQTTALPDEDDDL--- 216
Chlamy Ran1    VEEVALPPPEVQIDLAEQQRNEAELEQAAQQPLPADDDDELLD 223
               *   ** ****  :* *   : * :** *    ** :***
```

Figure S3. Sequence alignment of human Ran with *Chlamydomonas* Ran like small GTPase (Ran1).

Figure S4

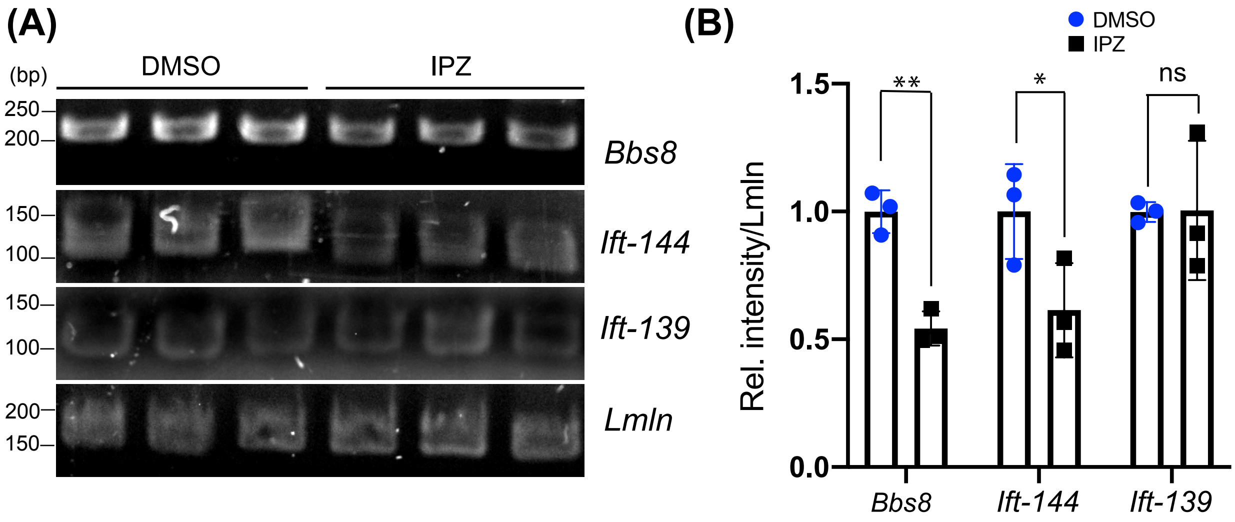

Figure S4. The small molecular inhibitor IPZ selectively inhibits the expression of cilia regrowth-associated genes

Figure S5

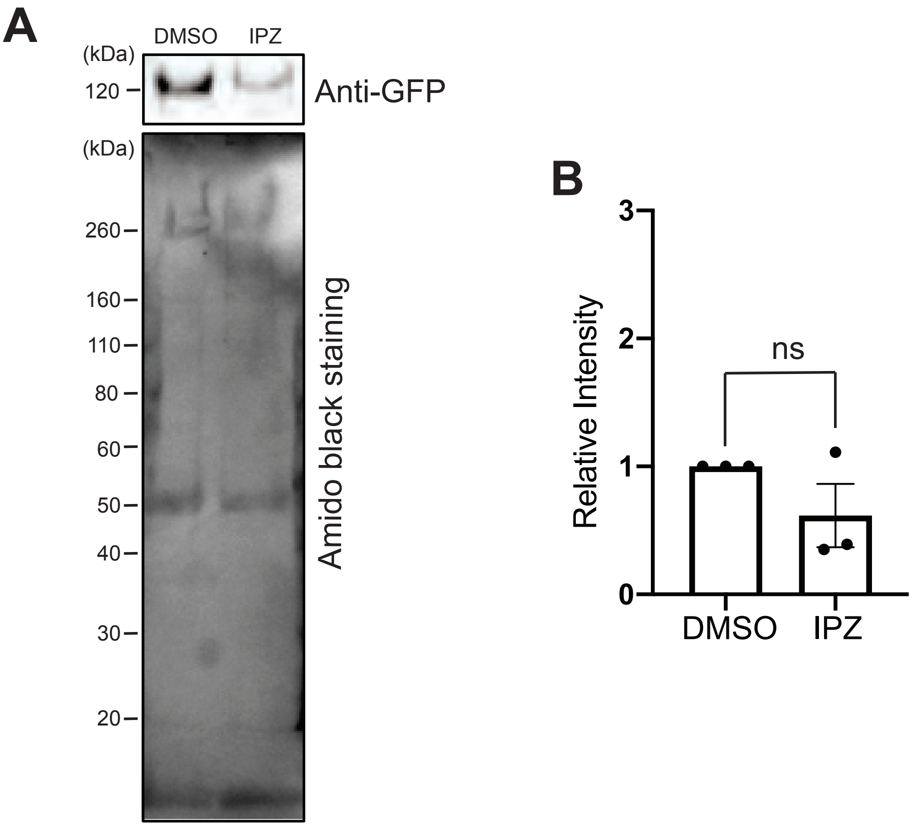

Figure S5. Immunobolt analysis of KAP-GFP protein levels in isolated *Chlamydomonas* cilia with IPZ treatment.

Figure S6

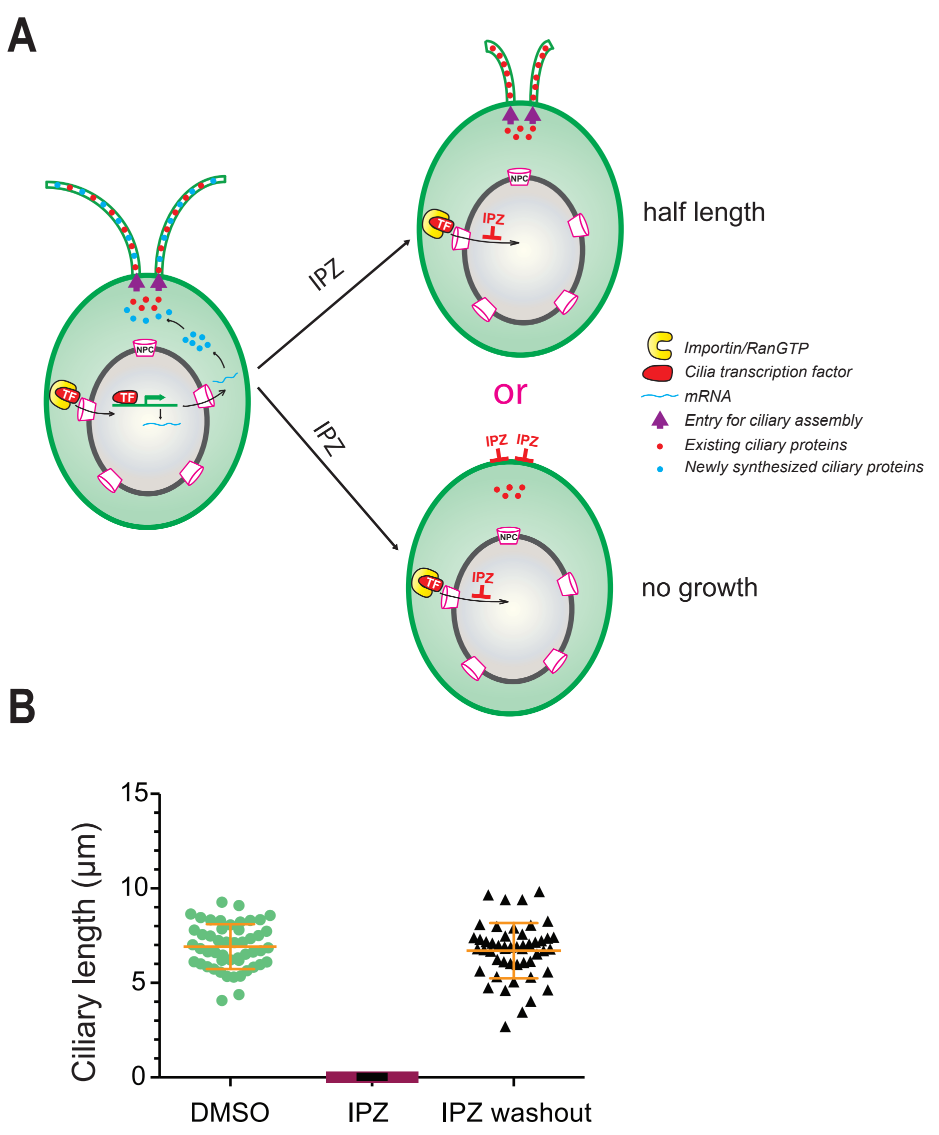

Figure S6. RanGTP regulates ciliary protein incorporation in *Chlamydomonas* regardless of the presence of new synthesized proteins.
